# Investigation of Processing Conditions and Product Geometry in Out-Mold Decoration and Their Effects on Film Adhesion and Deformation

**DOI:** 10.3390/polym17243239

**Published:** 2025-12-05

**Authors:** Hui-Li Chen, Po-Wei Huang, Sheng-Hsun Hsu, Jhong-Sian Wu

**Affiliations:** 1 Mechanical Engineering, College of Semiconductor Engineering, CTBC University of Technology, Tainan 74448, Taiwan; 2 Mechanical and Automation Engineering, College of Engineering, Taiwan Steel University of Science and Technology, Kaohsiung 821013, Taiwan; bowei8915@gmail.com; 3 Plastic Precision Machining Center, College of Semiconductor Engineering, CTBC University of Technology, Tainan 74448, Taiwan

**Keywords:** out-mold decoration processing, molded parts of 2.5D and 3D, product geometry, film adhesion, film deformation

## Abstract

The growing demand for high-quality decorative polymer surfaces has increased interest in Out Mold Decoration (OMD), yet the combined influence of processing conditions and product geometry on film adhesion and deformation remains insufficiently defined. This study establishes an integrated framework that connects OMD process parameters with geometry-dependent deformation behavior using polycarbonate films printed with an ink grid. Adhesion and surface quality were evaluated using 2.5D specimens, while 3D models with varied fillet radii, slopes, and heights enabled quantitative assessment of grid-spacing evolution and thickness distribution. Results show that preheating smooths the film without improving adhesion, whereas increasing the forming environment temperature enhances both bonding and surface quality within the material’s thermal tolerance. Vacuum pressure strengthens film–substrate contact but requires moderation to prevent overstretching. An optimized condition of 100 °C preheating, 90 °C forming temperature, and 2.5 kg vacuum pressure provides a balanced performance. Geometric factors exert strong control over deformation, with small radii, steep slopes, and tall features producing greater strain and nonuniform thinning. These findings establish practical processing windows and geometry guidelines for achieving reliable OMD components that integrate high visual quality with stable adhesion performance.

## 1. Introduction

In recent years, the demand for high-quality exterior surfaces in electronic products, automotive interiors, and household appliances has continued to rise, as consumers increasingly expect both functional performance and visually appealing designs [1,2,3,4,5,6]. To meet these demands, polymer-based decorative technologies have become an essential focus in both industrial applications and academic research. Previous studies examining embossed and textured injection-molded products have demonstrated that surface replication fidelity, gloss, and overall appearance are strongly governed by thermal gradients and mold-temperature control during forming [1]. These findings collectively highlight that achieving high-quality decorative surfaces is fundamentally linked to the interplay between material behavior and processing conditions. However, as product designs evolve toward more geometrically complex surfaces, conventional decoration techniques face growing challenges in maintaining surface uniformity, pattern stability, and aesthetic consistency.

As a result, surface-decoration technologies for polymer products have become a major focus in both industrial applications and academic research. Conventional decoration methods such as painting, post-coating, and in-mold decoration (IMD) have been widely employed to enhance gloss, texture, and tactile characteristics [7,8,9,10,11,12,13,14]. These approaches perform effectively for flat or low-curvature components; however, as product designs increasingly evolve toward complex three-dimensional shapes and large curved surfaces, limitations in conformity, durability, environmental impact, and visual consistency have become more apparent. Among existing techniques, IMD remains widely adopted because it integrates decorative films directly during injection molding, offering high productivity and stable performance for simple geometries. Nevertheless, IMD is inherently more suitable for flat or mildly curved surfaces, and when applied to components with high curvature, deep cavities, or complex geometries, insufficient film extensibility often results in wrinkling, tearing, or severe pattern distortion. Related investigations further demonstrate that injection-molding parameters such as melt temperature, injection speed, and mold temperature strongly influence film deformation and pattern stability, particularly when the film is subjected to shear-induced sliding or nonuniform extension during cavity filling [11,12]. Recent modeling studies also indicate that IMD film deformation can be predicted using constitutive models such as Johnson–Cook, highlighting that film mechanics, localized stress concentration, and thermal softening play essential roles in determining the stability and fidelity of decorative layers [13]. These findings collectively underline the fundamental limitations of IMD when dealing with complex topographies and reinforce the need for more flexible decoration technologies capable of accommodating greater geometric freedom and achieving higher aesthetic precision.

**Figure 1:**
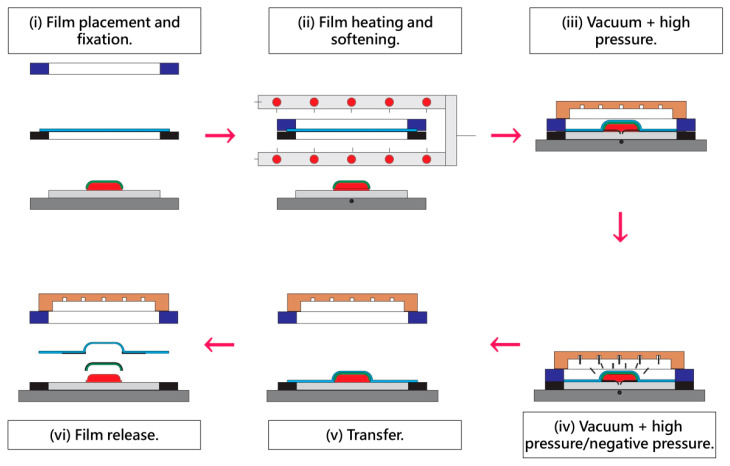
Out Mold Decoration (OMD) Film Processing Workflow.

In addition, IMD offers limited flexibility in optimizing local heating, pressure distribution, or compensation strategies, which restricts its adaptability to next-generation products that require precise formability and high geometric freedom. By contrast, Out Mold Decoration (OMD) separates film preparation from substrate molding, allowing independent control of film preheating, vacuum forming, and high-pressure transfer [8,15,16,17,18,19,20], as illustrated in Figure 1. This separation enables the film to conform more accurately to complex geometries and helps overcome the deformation challenges frequently observed in IMD. Prior studies have shown that film thickness, thermal response, and interfacial characteristics significantly influence deformation behavior [21,22], while research on polymeric film formation has further demonstrated that intrinsic film properties, including dispersion stability, microstructure evolution, and membrane morphology, directly affect optical appearance, mechanical uniformity, and susceptibility to thinning during forming [22,23]. These findings collectively indicate that surface-decoration quality depends on the combined effects of thermal and mechanical processes as well as the inherent material behavior of the film [24,25]. Nevertheless, existing literature has mainly examined IMD molding parameters or film-forming characteristics separately and lacks an integrated framework that connects controlled processing variables with deformation mechanisms across three-dimensional geometries. This research gap is highly relevant to the increasing industrial demand for high-precision decorative components used in consumer products and automotive interiors, where visual quality and long-term durability strongly influence user perception and market competitiveness. Therefore, a systematic investigation is needed to clarify how process parameters and product geometry jointly determine adhesion, surface quality, and deformation behavior in OMD forming so that more reliable and advanced decorative manufacturing technologies can be developed.

Building on these considerations, this study establishes a unified framework that clarifies how OMD processing parameters and product geometry collectively determine film adhesion, surface quality, and deformation outcomes. By employing 2.5D specimens and 3D models with systematically varied fillet radii, slopes, and heights, this work quantifies film behavior through peel testing, roughness measurement, grid-spacing evaluation, and thickness analysis. Unlike previous studies that examined IMD or OMD effects in isolation, this research provides the first integrated assessment linking processing conditions with complex three-dimensional geometries. This contribution fills a critical gap in current decoration-molding literature and delivers a scientific foundation for designing next-generation OMD processes capable of achieving reliable adhesion and stable forming on highly curved 3D surfaces.

## 2. Experiment Setups

### 2.1. Sample-Preparation

**Figure 2:**
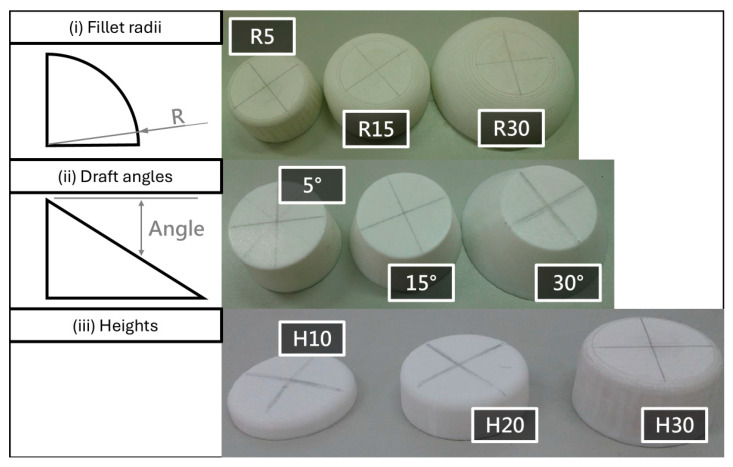
OMD-formed 3D specimens: (**i**) models with different fillet radii (**ii**) models with varying draft angles, and (**iii**) models with different heights.

In this study, two types of specimens were prepared to investigate the film adhesion strength and extensibility of OMD products: (i) tensile specimens conforming to ASTM D638 [26] (type 2.5) for evaluating the mechanical properties of the films, and (ii) three-dimensional (3D) models with systematically varied geometrical features. The 3D models were designed with different corner radii (R5, R15, R30), draft angles (5°, 15°, 30°), and heights (H10, H20, H30), as illustrated in Figure 2. This experimental design enabled a comprehensive evaluation of how geometric factors influence film deformation and adhesion performance.

### 2.2. Equipment

In this study, the post-forming state of decorative films in the out-mold decoration (OMD) process was investigated using a high-pressure transfer molding machine (VF-200TON, IMF Technology Co., Ltd., Taipei, Taiwan). The OMD operation involved sequential steps of film preheating, vacuum application, and high-pressure forming (Figure 1ii–iv). Furthermore, to evaluate the stability of decorative patterns under such deformation, commercially produced polycarbonate (PC) films (sup-plied by Jin Taiwan Enterprise Co., Ltd., Tainan City, Taiwan) were screen-printed with an ink grid prior to forming. The films possessed a thickness of 0.5 mm and planar dimensions of 100 mm × 100 mm, and were screen-printed with an ink grid prior to forming. The selected polycarbonate film thickness of 0.5 mm was deemed optimal as it provides a balanced combination of formability, structural stability, and measurement reliability required for OMD experiments. A thinner film would exhibit higher extensibility but would also be more prone to excessive thinning, wrinkling, or unintended tearing during vacuum forming, making deformation risks difficult to control. Conversely, a thicker film would possess greater stiffness, reducing its ability to conform to complex 3D geometries and making grid-spacing or thickness-variation analysis less sensitive to process-induced deformation. The 0.5 mm thickness, therefore, represents an optimal intermediate value that ensures stable forming behavior, prevents premature failure, and allows deformation, elongation, and pattern distortion to be quantitatively evaluated with high repeatability under typical OMD forming conditions. The ink used in this study was supplied by Proell Services GmbH (Hildesheim, Germany). Formulated with resin-based components, the ink was specifically designed to accommodate film deformation during OMD, thereby preventing defects such as cracking or washout caused by temperature variations or elongation. It also exhibited excellent adhesion and abrasion resistance. For the experiments, the NORIPHAN HTR series was adopted, with the black color designated as HTR952, combined with the diluent HTR090. The diluent functioned to accelerate drying and curing (mixing ratio of ink diluent of 10∶1). In addition, functional additives were introduced to further enhance the dry resistance and long-term stability of black ink.

### 2.3. Methodology

**Figure 3:**
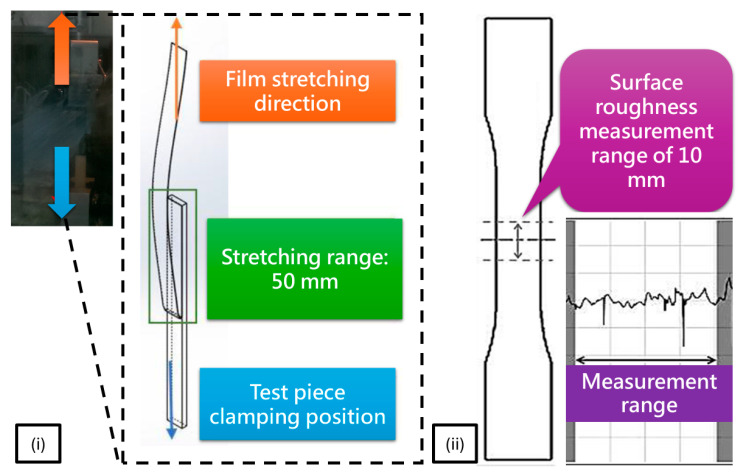
Measurement Procedure for 2.5D Formed Specimens: (**i**) Adhesion strength test between film and specimen; (**ii**) Surface roughness measurement points on the film.

In this study, a systematic experimental methodology was established to investigate film–substrate adhesion behavior, surface quality, and film extensibility during the out-mold decoration (OMD) process. Both 2.5D and 3D specimens were designed to provide complementary insights into film deformation and interface characteristics under controlled and practical forming conditions. For the 2.5D specimens, the experiments primarily focused on quantifying the adhesion performance through a film peel test and evaluating the evolution of surface roughness after forming (Figure 3). The specific processing conditions applied in this phase, including different film preheating temperatures, different forming environment temperatures, and different vacuum forming pressures, are summarized in Table 1.

During peel testing, the film was mechanically separated from the substrate under a controlled displacement rate following the procedure outlined in the standard peel test (ASTM D903 [27]). This approach enabled direct measurement of the interfacial resistance and provided a realistic representation of the debonding behavior that may occur during OMD processing. The clamping configuration was designed to maintain a fully constrained and stable peeling path, ensuring uniform load transfer and minimizing slippage throughout the test (Figure 3i). The peel test was conducted by separating the film from the substrate at a constant 180° angle under a displacement-controlled loading rate of 20 mm/min, with a total upward displacement of 50 mm. The peel strip was trimmed to a width of 13 mm, corresponding to the designated specimen width used in this study. To assess changes in surface quality, roughness measurements were performed at predefined locations on the film surface, with a measurement length of 10 mm, as shown in Figure 3ii. An instrument for measuring surface roughness (SJ-310, Mitutoyo, Taichung, Taiwan) was used to obtain Ra values with high precision. These measurements enabled detailed characterization of surface deviations induced by stretching, thermal softening, and localized strain of the film, thereby providing insights into how processing conditions influence surface integrity and post-forming appearance.

To ensure that the evaluation of film adhesion and roughness variation distribution accurately reflects the influence of processing conditions, all measurements in this study were performed under strictly controlled and repeatable procedures. For each forming condition and each geometric model, five specimens were randomly selected to eliminate sampling bias, and the corresponding average values and standard errors were calculated to ensure statistical reliability. Because the experimental setup employed standardized film preparation, consistent forming sequences, and calibrated measurement instruments, the measurement uncertainty is expected to be minimal. The detailed quantitative results are summarized in Table 2 and serve as the basis for subsequent analysis of peel-test and processing conditions.

**Figure 4:**
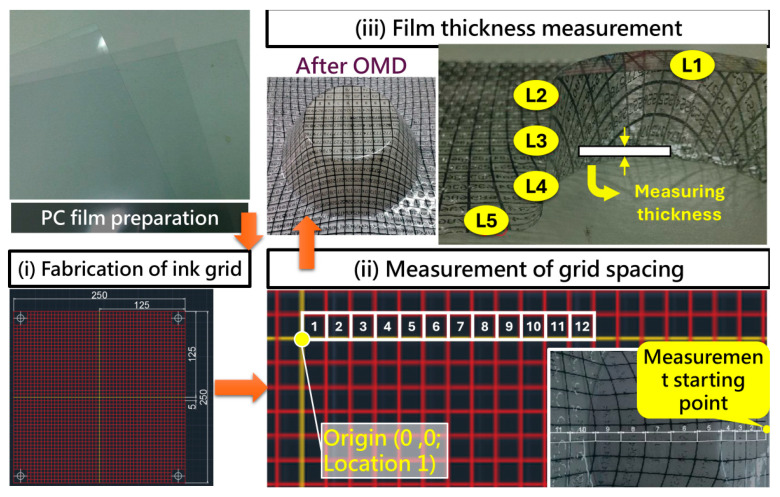
Preparation and Measurement Procedure for 3D Forming Films: (**i**) Fabrication of ink grid. (**ii**) Measurement of grid spacing. (**iii**) Film thickness measurement.

For the 3D forming tests, the focus was placed on the extensibility of the film and the stability of decorative patterns under geometric deformation. Figure 4 presents the complete procedure for preparing and evaluating 3D-formed films. PC films were first pretreated—this stage also included cutting and cleaning—to eliminate contamination or surface defects prior to printing and forming. Screen printing was then performed to fabricate a uniformly distributed ink grid on the film surface, as shown in Figure 4i. This grid served as a reference pattern for tracking deformation behavior under different curvatures and geometric features.

- After OMD forming, the spacing between the printed grid lines changed due to film stretching, which enabled quantitative assessment of strain distribution across different regions. The printed grid consisted of 50 cells, each measuring 5 × 5 mm, yielding an overall patterned area of 250 × 250 mm. This uniformly spaced grid provided stable and repeatable reference points for quantifying post-forming deformation, enabling precise evaluation of local strain and distortion across the film surface. As shown in Figure 4ii, grid-spacing measurements were performed sequentially along predetermined paths starting from a designated reference position. The actual spacing variations at each location were recorded using a precision vernier caliper (±0.02 mm resolution) to ensure accurate measurement of the deformed grid intervals. To determine deformation or strain, the measured grid spacing after forming was compared with the original undeformed grid spacing. The engineering strain for each grid segment was calculated as

(1) $$\varepsilon =\frac{∆L}{L_{0}}$$

where $L_{0}$ is the initial grid length and ∆L is the change after forming. This procedure enabled precise quantification of localized stretching and provided a reliable basis for analyzing geometry-induced film deformation behavior.

- These data were used to construct strain distribution maps of the film after forming, thereby enabling analysis of how geometric parameters such as fillet radius, slope, and height affected the uniformity of film extension. In addition, film thickness was measured at five specific positions (L1 to L5), as shown in Figure 4iii. These positions covered regions ranging from high-curvature areas to relatively flat surfaces. Thickness measurements were obtained using a digital thickness gauge (Model MIT-DTG-S, accuracy ±0.01 mm; SEAT Industrial Co., Ltd., Qianzhen District, Kaohsiung City, Taiwan) to ensure precise detection of thickness variation in different regions. By comparing thickness values across these locations, it was possible to determine whether localized thinning occurred due to stress concentration during deformation.
- Thickness variation (%) in this study is calculated based on the relative change in film thickness before and after OMD forming, using the following expression:

(2) $$Thickness\;Variation\;(\%)=\frac{t_{initial}-t_{measured}}{t_{measured}}\times 100$$

where $t_{initial}$ is initial PC film thickness (0.5 mm), and $t_{measured}$ is the film thickness at each measurement point after forming. Because the denominator is the initial thickness of only 0.5 mm, even a relatively small absolute thickness reduction (for example, 0.15–0.25 mm) corresponds to a large percentage change (30–50%). Measurement points L1 to L5 were selected along the forming direction of the film to capture deformation behavior under progressively increasing strain. L1 was positioned at the origin of the printed grid on the undeformed film and served as the baseline reference. L2 corresponded to the region between the fifth and sixth grid intersections, L3 to the region between the sixth and seventh intersections, L4 to the region between the seventh and eighth intersections, and L5 to the region between the tenth and eleventh intersections. Spatially, L1 is in the flat baseline region, L2, L3, and L4 lie within transitional curvature zones where moderate geometric influence occurs, and L5 corresponds to the area subjected to the highest geometric deformation. This coordinated layout provides a systematic mapping of film behavior from minimally strained regions to zones experiencing substantial extension, thereby enabling accurate evaluation of thickness variation across different deformations during OMD forming.

To ensure that the evaluation of elongation and grid-spacing variation/thickness distribution accurately reflects the influence of product geometry, all measurements in this study were performed under strictly controlled and repeatable procedures. For each forming condition and each geometric model, five specimens were randomly selected to eliminate sampling bias, and the corresponding average values and standard errors were calculated to ensure statistical reliability. Because the experimental setup employed standardized film preparation, consistent forming sequences, and calibrated measurement instruments, the measurement uncertainty is expected to be minimal. The detailed quantitative results are summarized in Table 3 and serve as the basis for subsequent analysis of deformation mechanisms and process–geometry interactions.

## 3. Experiment Results and Discussion

### 3.1. Effect of Different Processing Conditions on the OMD Films After Forming

**Figure 5:**
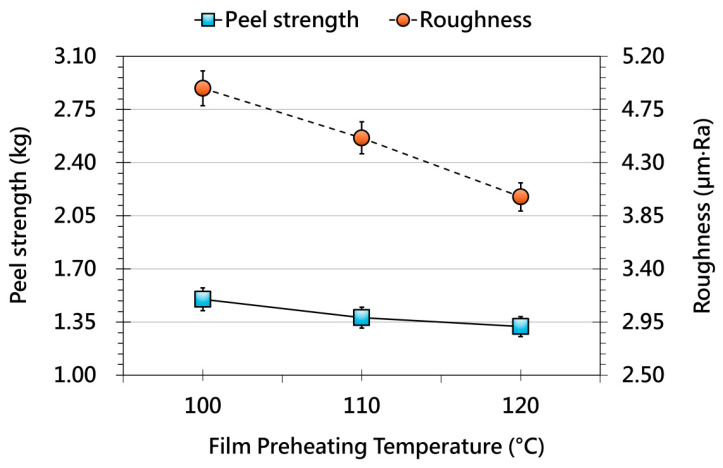
Effect of different film preheating temperatures on the peel strength (solid line, primary y-axis) and surface roughness (dashed line, secondary y-axis) of OMD films after forming.

**Figure 6:**
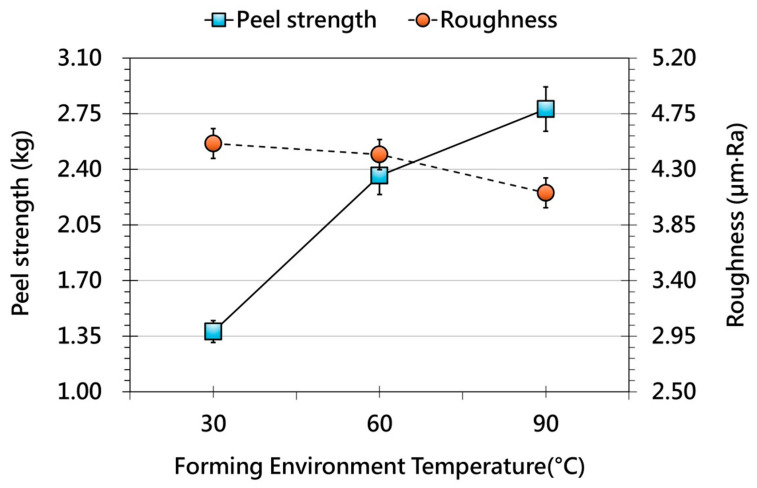
Effect of different forming environment temperature on the peel strength (solid line, primary y-axis) and surface roughness (dashed line, secondary y-axis) of OMD films after forming.

Figure 5 illustrates the influence of film preheating temperature on OMD film behavior after forming. As the preheating temperature increases, adhesion strength remains largely unchanged or shows a slight decline, suggesting that thermal softening alone is insufficient to enhance interfacial bonding. This behavior may result from surface relaxation of the film, which reduces microstructural features responsible for mechanical interlocking. In contrast, surface roughness consistently decreases with higher preheating temperatures, indicating that moderate heating improves film flow and conformity to the mold surface. This demonstrates a clear trade-off in which elevated preheating enhances surface quality but does not reinforce adhesion. Figure 6 presents the effect of forming-environment temperature. A distinct upward trend in adhesion strength is observed as environmental temperature increases, likely attributable to enhanced molecular mobility and improved film–substrate contact. Simultaneously, surface roughness decreases, demonstrating that environmental heating promotes uniform deformation and smoother surface formation. However, temperatures beyond the thermal tolerance of the film or printed layer may cause thermal degradation or visual distortion, indicating that environmental temperature must be carefully optimized.

**Figure 7:**
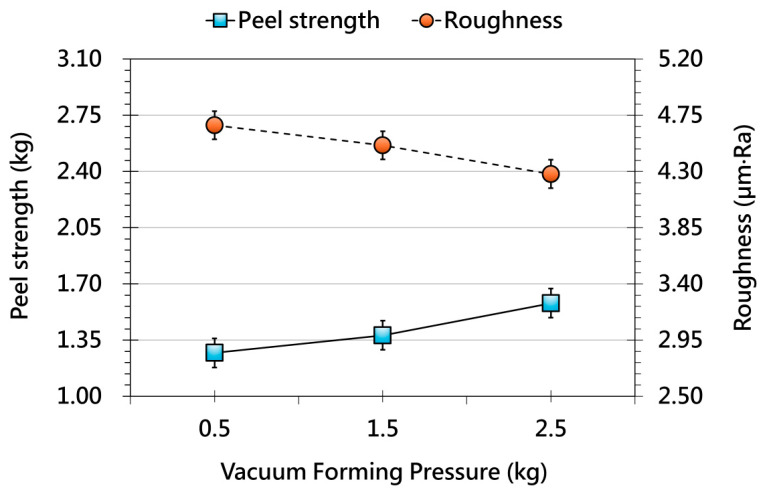
Effect of different vacuum forming pressure on the peel strength (solid line, primary y-axis) and surface roughness (dashed line, secondary y-axis) of OMD films after forming.

**Figure 8:**
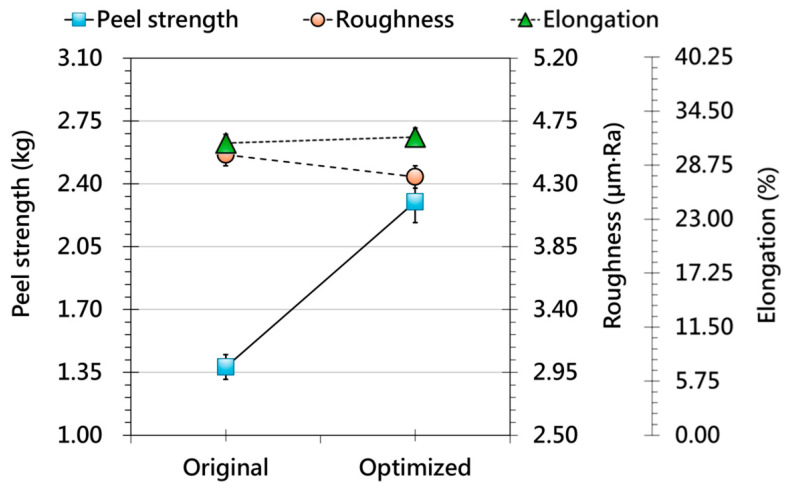
Comparison of OMD film responses under original and optimized processing conditions, including peel strength (solid line, primary y-axis), surface roughness (dashed line, secondary y-axis), and grid-spacing variation (dotted line, secondary y-axis) after forming.

Figure 7 shows the effect of vacuum forming pressure. Increasing pressure improves adhesion strength by promoting more intimate film–substrate contact and reducing interfacial voids. Surface roughness also decreases steadily, reflecting improved film conformance during forming. While vacuum pressure is a key factor in achieving high adhesion and surface quality, excessive pressure may lead to overstretching, thickness nonuniformity, or hidden microcracks. These results highlight the need for a balanced pressure window that maximizes interfacial bonding without compromising mechanical stability. Synthesizing the above trends, an optimized OMD processing condition is identified at 100 °C film preheating, 90 °C forming environment temperature, and 2.5 kg vacuum pressure, representing the best balance between adhesion strength and surface uniformity. Figure 8 compares the optimized condition with the original parameters and shows pronounced improvements in adhesion, reduced roughness, and more stable elongation behavior. These results confirm that multi-parameter coordination—rather than adjustment of a single variable—is essential for achieving high interface reliability and consistent surface quality. The optimized setting therefore provides a practical reference for industrial OMD processes. In the second phase of this study, these optimized parameters are applied to 3D geometries to systematically analyze film deformation as it transitions from 2.5D to 3D, ensuring consistent evaluation without process-related interference.

### 3.2. Effect of Different Product Geometry on the OMD Films After Forming

**Figure 9:**
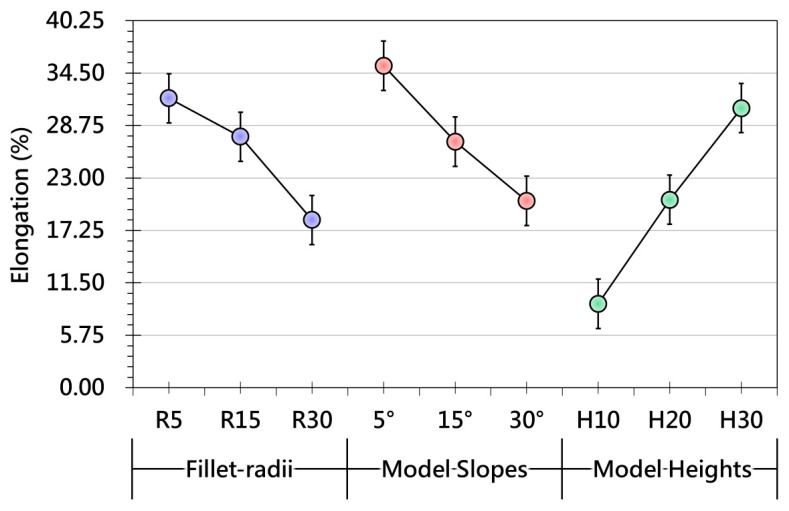
Effects of product geometry, including variations in fillet radius, slope, and height, on the grid-spacing distribution of OMD films after forming.

**Figure 10:**
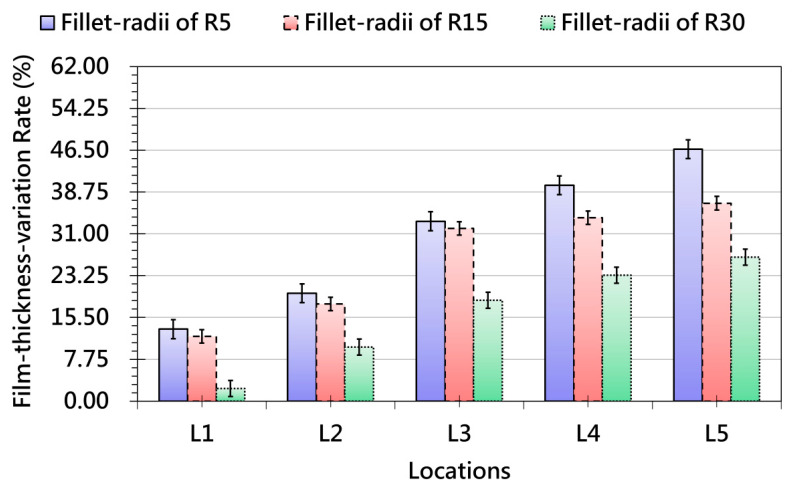
Thickness-variation Trends Measured at Different Locations after OMD Film Formation-due to Different Fillet-radii.

Figure 9 presents the trend of grid-spacing variation as influenced by different geometric features of the product. When the fillet radius is small, the film experiences concentrated local stretching, leading to larger changes in grid spacing. As the radius increases, curvature becomes smoother, reducing stress concentration and resulting in more uniform deformation. A similar geometric effect is observed for slopes: gentle slopes allow the film to conform gradually with limited grid-spacing variation, whereas steeper slopes trigger sharp increases in deformation, particularly in regions of high three-dimensional curvature. Model height further amplifies this behavior. Taller features require the film to accommodate greater vertical displacement, resulting in significantly larger elongation and pronounced grid-spacing changes. Overall, Figure 9 demonstrates that small radii, steep slopes, and tall heights all intensify deformation demand and directly influence the stability of surface pattern distribution. In addition, Figure 10 examines thickness variation under different fillet radii and further confirms the dominant role of curvature in local deformation behavior. Small fillets generate higher thickness variation across most regions because sharp corners amplify mechanical stress and induce localized thinning. As the radius increases, stress is more evenly distributed and thickness variation decreases, with measurement points converging toward a more stable profile. Regions near geometric transitions continue to exhibit greater fluctuation, highlighting the geometric sensitivity of the forming process.

**Figure 11:**
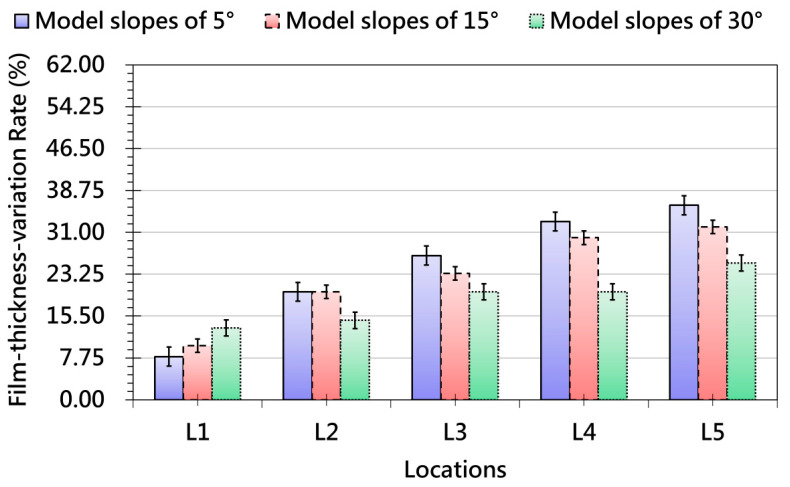
Thickness-variation Trends Measured at Different Locations after OMD Film Formation-due to Different Model Slopes.

**Figure 12:**
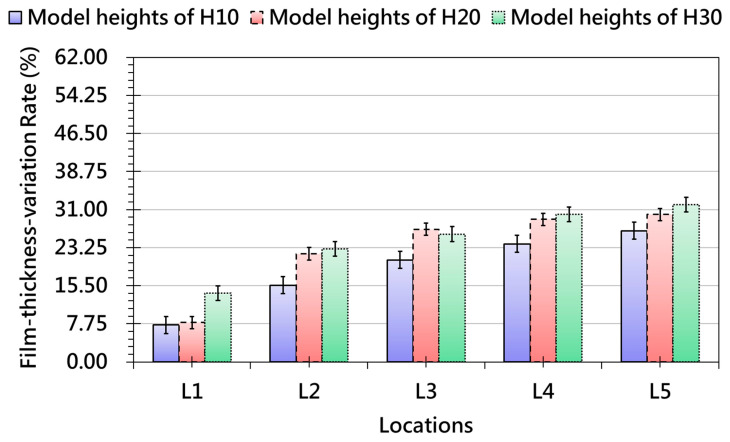
Thickness-variation Trends Measured at Different Locations after OMD Film Formation-due to Different Model Heights.

Figure 11 shows that slope angle has a strong and nonlinear effect on thickness uniformity. Small slopes promote gradual deformation and maintain low variation, while steeper slopes generate substantial tensile stresses, particularly in transition zones where curvature gradients intensify strain transfer. Thickness variation peaks near the top and base of the slope, while mid-sections remain relatively stable, demonstrating that strain redistribution during forming is highly dependent on local geometry. These results highlight the inherent trade-off in slope design: increased geometric freedom comes with a higher risk of non-uniform thinning. Figure 12 reveals that model height further magnifies deformation instability. Low-height models produce relatively uniform film thickness, but as height increases, the film must stretch across a greater distance, leading to progressive increases in localized thinning. Severe variation occurs near elevated features, while flatter regions remain unaffected, confirming that height is a critical driver of film-forming non-uniformity.

Collectively, Figure 9, Figure 10, Figure 11 and Figure 12 demonstrate that fillet radius, slope, and height act as key geometric parameters governing strain distribution, grid-spacing stability, and thickness uniformity in OMD. Sharp radii, steep slopes, and tall features consistently intensify local strain, making them high-risk regions for pattern distortion and thinning-induced defects. These insights provide practical guidance for OMD process design, emphasizing that optimized forming strategies—such as controlled preheating, proper vacuum pressure, and localized thermal management—are essential to ensure stable deformation, strong adhesion, and high-quality surface appearance in products with complex 3D geometries.

## 4. Conclusions

This study established an integrated understanding of how processing parameters and product geometry jointly govern film adhesion, surface quality, and thickness uniformity in OMD. By decoupling the roles of film preheating, forming environment temperature, and vacuum forming pressure, and then re-evaluating geometric effects under the optimized setting, we showed that robust interfacial bonding and stable morphology arise from coordinated multi-parameter tuning rather than from isolated adjustments. The resulting guidelines connect process windows with geometric sensitivities and provide actionable directions for industrial OMD design and scaling. The following points should be noted:

- This study demonstrated that the three key processing parameters—film preheating temperature, forming environment temperature, and vacuum forming pressure—exert distinct and non-linear effects on adhesion and surface quality. Increasing preheating temperature smooths the film but does not enhance adhesion, whereas higher environment temperature and vacuum pressure improve bonding and surface finish within safe operating limits.
- An optimized OMD processing window was identified at a film preheating temperature of 100 °C, a forming environment temperature of 90 °C, and a vacuum pressure of 2.5 kg. This coordinated setting yields superior adhesion, reduced roughness, and more stable elongation behavior.
- Geometric factors including fillet radius, slope angle, and model height play a decisive role in film deformation. Small radii, steep slopes, and tall features intensify strain, enlarge grid-spacing changes, and increase thickness variation.
- Combined measurements of grid spacing and film thickness reveal consistent deformation hotspots at curvature transitions and elevated features, confirming that both global elongation and local strain accumulation govern OMD film behavior.
- The integration of processing-parameter control with geometric considerations establishes a practical framework for designing OMD components with high adhesion, uniform deformation, and improved visual quality, enabling more reliable decoration of complex 3D products.

## Figures and Tables

**Table 1:** Configuration of the Processing Parameters Applied in OMD Film Forming Tests.

Method	Film Preheating Temperatures (°C)	Forming Environment Temperature (°C)	Vacuum Forming Pressure (kg)
1	100	60	1.5
2	110	60	1.5
3	120	60	1.5
4	100	30	1.5
5	100	60	1.5
6	100	90	1.5
7	100	60	0.5
8	100	60	1.5
9	100	60	2.5

**Table 2:** Experimental Measurements of Film Peel Strength and Surface Roughness under Different OMD Processing Conditions.

Film Preheating Temperatures (°C)	Peel Strength (kg)		Roughness (µm·Ra)	
	AVG.	ANOVA	AVG.	ANOVA
100	1.51	0.280%	4.93	0.280%
110	1.38	0.292%	4.51	0.292%
120	1.32	0.323%	4.01	0.322%
Forming Environment Temperature (°C)	Peel strength (kg)		Roughness (µm·Ra)	
	AVG.	ANOVA	AVG.	ANOVA
30	1.38	0.337%	4.51	0.337%
60	2.36	0.354%	4.42	0.354%
90	2.78	0.359%	4.11	0.359%
Vacuum Forming Pressure (kg)	Peel strength (kg)		Roughness (µm·Ra)	
	AVG.	ANOVA	AVG.	ANOVA
0.5	1.27	0.305%	4.67	0.305%
1.5	1.38	0.307%	4.51	0.307%
2.5	1.58	0.361%	4.28	0.361%

**Table 3:** Experimental measurements of film elongation and thickness variation under different product geometry configurations in OMD forming.

Fillet- radii	Elongation (%)		Film-Thickness-Variation Rate (%)									
			L1		L2		L3		L4		L5	
	AVG.	ANOVA	AVG.	ANOVA	AVG.	ANOVA	AVG.	ANOVA	AVG.	ANOVA	AVG.	ANOVA
R5	31.74	0.280%	13.3	0.292%	20.5	0.292%	33.3	0.290%	40.1	0.292%	46.7	0.272%
R15	27.52	0.292%	12.2	0.294%	18.4	0.294%	32.2	0.296%	34.2	0.298%	36.7	0.292%
R30	18.39	0.323%	2.3	0.332%	10.2	0.327%	18.7	0.344%	23.3	0.323%	26.7	0.310%
Model Slopes	Elongation (%)		Film-thickness-variation Rate (%)									
			L1		L2		L3		L4		L5	
	AVG.	ANOVA	AVG.	ANOVA	AVG.	ANOVA	AVG.	ANOVA	AVG.	ANOVA	AVG.	ANOVA
5°	35.31	0.337%	8.1	0.339%	20.8	0.339%	26.7	0.336%	33.1	0.337%	36.1	0.321%
15°	26.99	0.354%	10.1	0.356%	20.4	0.356%	23.4	0.356%	30.2	0.297%	32.2	0.348%
30°	20.5	0.359%	13.3	0.361%	14.7	0.360%	20.7	0.359%	20.3	0.352%	25.3	0.377%
Model Heights	Elongation (%)		Film-thickness-variation Rate (%)									
			L1		L2		L3		L4		L5	
	AVG.	ANOVA	AVG.	ANOVA	AVG.	ANOVA	AVG.	ANOVA	AVG.	ANOVA	AVG.	ANOVA
H10	9.21	0.305%	7.5	0.318%	15.6	0.318%	20.8	0.340%	24.2	0.297%	26.7	0.300%
H20	20.62	0.307%	8.2	0.309%	22.6	0.309%	27.6	0.313%	29.4	0.316%	30.1	0.295%
H30	30.67	0.361%	14.2	0.364%	23.1	0.364%	26.2	0.368%	30.6	0.363%	32.2	0.350%

## Data Availability

The original contributions presented in this study are included in the article. Further inquiries can be directed to the corresponding author.
